# Dual antiplatelet instead of intravenous thrombolysis for minor nondisabling acute ischemic stroke: A perspective from China

**DOI:** 10.2478/jtim-2023-0138

**Published:** 2024-03-21

**Authors:** Yu Cui, Xiao-Qiu Li, Hui-Sheng Chen

**Affiliations:** Department of Neurology, General Hospital of Northern Theater Command, Shenyang, 110016, Liaoning, Province, China

**Keywords:** dual antiplatelet, intravenous thrombolysis, minor stroke

Stroke was one of the most frequent diseases with significant disability and mortality.^[1]^ Several studies confirmed that intravenous thrombolysis with alteplase can effectively improve the functional outcomes in patients with acute ischemic stroke.^[2,3]^ Hence, intravenous thrombolysis with alteplase is recommended according to the current guideline for the management of patients with acute ischemic stroke presenting within 4.5 h of symptom onset.^[4]^ Minor stroke, defined as a National Institutes of Health Stroke Scale (NIHSS) score less than or equal to 3 or 5, accounted for approximately half of patients with acute ischemic stroke.^[5,6]^ Despite the mild nature of the neurological deficit in minor stroke, there exists a potential risk for developing disability as a result of stroke progression, underappreciated neurologic deficits (such as isolated limb numbness), or concurrent medical comorbidities. The attention of clinical physicians in the field has been drawn to minor strokes that do not result in clear disabling deficits, except for cases where the stroke does cause minor disability. The efficacy of intravenous thrombolysis in this population is still a subject of debate.^[7]^ It has often been assumed that the potential benefits of intravenous thrombolysis would be outweighed by the risks for individuals with minor stroke symptoms. A study conducted in Canada revealed that 28.5% of patients with minor stroke who did not receive intravenous alteplase treatment were unable to walk independently upon discharged.^[8]^ According to the current guideline, intravenous alteplase is recommended for patients with acute disabling mild ischemic stroke within 4.5 h of symptom onset.^[4]^ However, patients with nondisabling stroke symptoms (NIHSS score 0 to 5) are not recommended to receive intravenous alteplase within the same time frame. This recommendation is based on the Potential of rtPA for Ischemic Strokes With Mild Symptoms (PRISMS) trial, which was prematurely terminated due to the sponsorship reason.^[9]^ Approximately one third of patients did not benefit from intravenous alteplase, ^[10,11]^ thereby leaving the optimal treatment for acute minor ischemic stroke uncertain.

In June 2023, the results of Antiplatelet *vs*. R-tPA for Acute Mild Ischemic Stroke (ARAMIS) trial were published in the Journal of American Medical Association.^[12]^ The trial findings demonstrated that dual antiplatelet treatment (DAPT) consisting of aspirin and clopidogrel was noninferior to intravenous alteplase in terms of achieving excellent functional outcome at 90 days among patients with minor nondisabling acute ischemic stroke presenting within 4.5 h of symptom onset. These results provided robust evidence supporting the efficacy of DAPT, while also highlighting its superior safety profile in this population. The finding was consistent with a prospective study based on Austrian Stroke Unit Registry study, which indicated that intravenous thrombolysis was not superior to DAPT in the treatment of mild non-cardioembolic stroke and may potentially have adverse effects.^[13]^ Furthermore, this finding was presented at the International Stroke Conference 2023 and garnered significant attention. The PRISMS trial previously reported that among patients with minor nondisabling acute ischemic stroke, treatment with alteplase compared with aspirin did not result in a higher likelihood of favorable functional outcome at 90 days.^[9]^ Despite the premature termination of the PRISMS trial precluded any definitive conclusions, the disparities observed between the PRISMS and ARAMIS trials have afforded us an opportunity to provide valuable insights into the utilization of DAPT in the context of acute nondisabling minor ischemic stroke from a Chinese perspective.

## Aramis versus prisms

The PRISMS trial, the first randomized clinical trial to assess the effect of antiplatelet and intravenous alteplase in disabling minor stroke, yielded inconclusive findings due to premature termination. Consequently, the current guideline does not endorse the use of intravenous alteplase for minor non-disabling stroke, as indicated by the results of PRISMS trial.^[4]^ In contrast, the ARAMIS trial provided evidence supporting the noninferiority of DAPT compared to intravenous alteplase. The ARAMIS trial and the PRISMS trial differed primarily in terms of the strength of antithrombotic treatment. In the PRISMS trial, participants received aspirin alone at a dosage of 325 g alone for a duration of 90 days. In contrast, the ARAMIS trial utilized a combination of aspirin and clopidogrel for a period of 10–14 days, followed by guideline-based antiplatelet treatment until reaching 90 days. The selection of DAPT was based on the findings of Clopidogrel in High-Risk Patients With Acute Non-Disabling Cerebrovascular Events (CHANCE) and Platelet-Oriented Inhibition in New TIA and Minor Ischemic Stroke (POINT) trial.^[14,15]^ The CHANCE and POINT trials demonstrated that in the individuals with transient ischemic attack or minor stroke who were eligible for treatment within 12–24 h after symptoms onset, the utilization of a combination of clopidogrel and aspirin was superior to aspirin alone in reducing the risk of ischemic events within a 90-day period. Furthermore, both trials observed comparable rates of hemorrhagic stroke or bleeding events. Despite the slight variation in the definition of minor stroke between the CHANCE or POINT trial (NIHSS score 0–3) and the ARAMIS trial (NIHSS score 0–5), a meta-analysis has demonstrated the superiority of DAPT over aspirin monotherapy in patients with minor stroke or high-risk transient ischemic attack, irrespective of the NIHSS score for minor stroke.^[16]^ The determination of the loading dose of antiplatelet drug is of utmost importance in ensuring safety. In the ARAMIS trial, the clopidogrel loading dose of DAPT treatment strategy (300 mg) was found to be comparable to that used in the CHANCE trial (300 mg), while differing from the dosage utilized in the POINT trial (600 mg). This discrepancy in dosages can be attributed to the similar population between the CHANCE and ARAMIS trials. Furthermore, a secondary analysis of the CHANCE trial indicated that the efficacy of reducing recurrent stroke with DAPT was observed within the initial two weeks.^[17]^ Moreover, a meta-analysis suggested that discontinuation of DAPT within 21 days, and potentially as early as 10 days, after initiation was expected to optimize advantages and mitigate potential harms.^[18]^ Consequently, in the ARAMIS trial, a 2-week DAPT comprising clopidogrel and aspirin was chosen to evaluate the effectiveness in comparison to intravenous alteplase.

## Advantage

According to current guideline, antithrombotic therapy should not be administered within 24 h after intravenous thrombolysis.^[4]^ This recommendation has posed a challenge for clinical physicians when managing early neurological deterioration caused by stroke progression, particularly in patients with minor stroke. The ARAMIS trial revealed that patients who received intravenous alteplase had a higher risk of experiencing early neurological deterioration compared to those who underwent DAPT.^[12]^ The results may be attributed to the efficacy of DAPT in preventing the progression of thrombus or the reoccurrence of stroke within 24 h of symptom onset. Conversely, patients who received intravenous alteplase exhibited a higher risk of experience early neurological deterioration due to short half-life of alteplase’s effect and the absence of antithrombotic treatment within the first 24 h following intravenous thrombolysis. The occurrence of early neurological deterioration following intravenous thrombolysis in acute ischemic stroke was found to be associated with an unfavorable prognosis.^[19]^ Therefore, in terms of mitigating the occurrence of early neurological deterioration, DAPT proved to be more suitable compared to intravenous thrombolysis due to its non-inferior impact on achieving excellent functional outcome at 90 days.

In the ARAMIS trial, as expected, patients receiving intravenous alteplase would experience higher rates of safety outcomes, such as symptomatic intracerebral hemorrhage and any bleeding events. Given the negative impact of symptomatic intracerebral hemorrhage on prognosis following intravenous thrombolysis,^[20]^ a lower incidence of bleeding events may lead to a more favorable prognosis in these patients. Overall, the findings of the ARAMIS trial endorsed the appropriate use of DAPT in patients with nondisabling minor stroke given the superior safety profile compared to intravenous alteplase.

## Concerns

Based on the finding of the ARAMIS trial, two categories of minor stroke patients warranted further investigation. The first category was patients with wake-up stroke or stroke of unknown onset. The WAKE-UP trial demonstrated that patients with favorable MRI findings who received intravenous alteplase treatment exhibited significantly improved functional outcomes. However, it was important to note that the median of NIHSS score of patients included was 6 (range from 4 to 9).^[21]^ The efficacy of intravenous thrombolysis in wake-up stroke has been established through advanced neuroimaging selection,^[22]^ however, the specific investigation of its efficacy in wakeup stroke with minor symptoms is lacking. Considering the strong correlation between onset-to-thrombolysis time and hemorrhagic transformation after intravenous alteplase, as well as the uncertain onset time in wake-up stroke,^[23]^ it is plausible to consider wake-up stroke with minor symptoms as a potential target for DAPT. The second was patients with nondisabling minor symptoms and large vessel occlusion (LVO) who may potentially benefit from DAPT. Previous studies have indicated the potential advantages of intravenous thrombolysis in patients with mild stroke symptoms and large artery atherosclerosis,^[24]^ however, the evidence is not robust. Furthermore, the benefits of intravenous thrombolysis have not been investigated in certain subgroups of minor stroke patients, such as those with tandem proximal intracranial occlusion and cervical internal artery lesion, basilar artery occlusion, and middle cerebral artery-M2 segment occlusion.^[25]^ Considering the limited efficacy of intravenous thrombolysis in achieving recanalization and the potential advantages of DAPT in cases of stroke with LVO, it is worth to exploring the comparative impact of DAPT versus intravenous alteplase in this specific population.

However, there were still some concerns about the findings in the ARAMIS trial. First, it is imperative that the noninferiority of this trial be established by utilizing DAPT as the control group, as the current guideline recommended DAPT for minor stroke cases.^[4]^ Nevertheless, the ambiguous advantages of DAPT on the 90-day mRS score and increasing percentage of patient receiving intravenous alteplase rendered the current design important to inform the best treatment. Second, previous studies have demonstrated the possible advantages of intravenous alteplase in patients with mild stroke with large artery atherosclerosis or large artery occlusion. Unfortunately, there were lower proportion of patients with vessel imaging test in the ARAMIS trial. Consequently, it is imperative to investigate the impact of DAPT and intravenous alteplase on patients exhibiting varying different degrees of vessel lesions. This would significantly enhance the generalizability of the findings. Third, there was a growing competition between the recombinant plasminogen activator tenecteplase and the established gold standard alteplase.^[26]^ The tenecteplase showed noninferior efficacy to alteplase and possessed advantages in terms of ease of use. However, it remained uncertain whether DAPT was also noninferior to intravenous tenecteplase. Fourth, it is widely recognized that hypertension is associated with the occurrence of early neurological deterioration following acute ischemic stroke,^[27]^ which can be predominantly prevented by the implementation of DAPT. Although previous study investigated the effect of hypertension management on prognosis following intravenous thrombolysis,^[28]^ up to date, there was no study exploring its effect on DAPT. Consequently, it is imperative to investigate the potential influence of effective hypertension management on the efficacy of DAPT in the future. Finally, further investigation is necessary to ascertain the benefits of DAPT in cases of minor stroke with large artery atherosclerosis etiology.

In conclusion, the utilization of DAPT should be highly recommended for the management of minor nondisabling acute ischemic stroke due to its convenient administration, cost-effectiveness, and favorable safety profile. In the future, the benefit of DAPT needed to be further confirmed in patients with wake-up stroke or LVO, thereby facilitating the implementation of individualized treatment.
